# Boosting laccase production by *Penicillium commune* s6 via solid-state fermentation on phenolic agri-wastes: statistical enhancement and bioremediation application in dye decolorization

**DOI:** 10.1186/s12896-026-01108-2

**Published:** 2026-03-11

**Authors:** Mohamed Abdelraof, Ghada S. A. Abdel Karim, Alshaimaa M. Elsayed, Abdelmageed M. Othman

**Affiliations:** 1 Microbial Chemistry Department, Biotechnology Research Institute, National Research Centre, Dokki, Giza 12622 Egypt; 2 Molecular Biology Department, Biotechnology Research Institute, National Research Centre, Dokki, Giza 12622 Egypt; 3 Faculty of Biotechnology, German International University, Regional Ring Road, East Cairo, New Administrative Capital, Egypt

**Keywords:** Laccase, Phenolic-rich food residues, *Penicillium commune*, Optimization, Bio-decolorization

## Abstract

**Background:**

Biological approaches, which involve applying the entire microbial cell or its released enzymes, provide an effective substitute for traditional techniques. By employing statistical optimization methodologies, key fermentation parameters can be precisely tuned to maximize enzyme yield.

**Results:**

*Penicillium commune* S6, a fungal isolate useful for laccase synthesis in solid-state fermentation employing a variety of phenolic-rich agri-food wastes, was identified by 18 S rRNA sequencing. By applying potato peel waste as solid-state fermentation substrate for 30 days of incubation at 30 °C, pH 8.0, 60% moisture content, and 4 mM CuSO_4_ using the inoculum size of 7.5%, *P. commune* S6 reached its maximal laccase production level, as determined by the one factor at a time (OFAT) technique. Laccase production would reach 6.20 U/gds at 2.71 mM CuSO_4_, 46 days of incubation, 12.64 g/flask of PPW content, and pH 9.13, according to response surface methodology (RSM) results. The laccase production levels predicted by the model and the actual experimental data showed a very good agreement, as indicated by the ANOVA analysis. *P. commune* S6 laccase was evaluated as a green alternative in dye decolorization, where it was found to be able to decolorize the Acid Dye Lanapel Red BM 143-PL at concentrations of 10, 25, and 50 mg L^− 1^ by 100, 64.08, and 36.86% after 120 min, which suggest that *P. commune* S6 laccase is likely useful in the biotechnological and industrial uses under milder circumstances.

**Conclusion:**

The integrated approach not only valorizes low-cost agro-industrial byproducts but also provides a robust, eco-friendly biocatalyst for wastewater treatment, highlighting a promising circular economy strategy for textile effluent detoxification.

**Supplementary Information:**

The online version contains supplementary material available at 10.1186/s12896-026-01108-2.

## Introduction

The industrial sector is very interested in microbial products, especially those that are based on enzymatic breakdown [1, 2]. Enzymes are ideal green biocatalysts because they operate efficiently under mild conditions with low energy input and produce minimal waste, aligning perfectly with the principles of sustainable development and green chemistry [3]. It is a promising option for use in several industrial domains because of its quick extraction process, reduced incubation duration, and abundance of different types of microbial enzymes [4]. Laccases are a widespread group of multicopper oxidases produced by various organisms [5]. Laccases catalyze the breakdown of diverse organic pollutants, including phenolics and amines, by oxidizing them while using water as the only byproduct from oxygen reduction [6–9]. This versatility has made laccases valuable biocatalysts across multiple industries, particularly in applications for bioremediation and environmental sustainability [10] such as degradation of polycyclic hydrocarbons [11], removal of micropollutants, removal of phenolic compounds [12], degradation of xenobiotics, and biosensors fabrication [13].

The cost of the growing cultural medium typically has a substantial impact on the fungal production of laccase, and the synthetic culture medium was mostly associated with poorer productivity and greater cost [14]. The utilization of agricultural peels as a culture medium represents an environmentally and economically advantageous strategy, aligning with the demand for high-output, low-cost microbial cultivation systems [15]. The wide availability and simple procurement of diverse crop peels make them a particularly viable option for formulating augmented media to enhance enzyme production [16]. There are usually a lot of nutrients in agri-food by-products, and these nutrients are frequently associated with lignin, cellulose, starch, and their derivatives [14]. Response surface methodology (RSM) has been widely used for the optimization of many biotechnological processes [8, 17, 18], as batch testing using established approaches might be challenging, costly, time-consuming, and massive [19]. This was achieved by skillfully optimizing the production conditions and culture medium components using a central composite design (CCD) statistical technique [8, 20].

The food, textile, plastics, and cosmetics sectors are just a few of the many businesses that use synthetic dyes extensively to display product colors. Over 10,000 different types of pigments and dyes are used in the printing and textile sectors, which led to the daily generation of enormous volumes of very polluted colored effluents [21]. According to statistics, the worldwide dye industry produces about 8 × 10^5^ tons of dye annually, and at least 10% of the dyes used in various industries end up in the environment and cause a range of ecological issues [22]. Chemical and physical procedures have historically been used to remove colors from wastewater, but they are ineffective, costly, and do not work with all types of dyes [23]. Biological approaches, which involve applying the entire microbial cell or its released enzymes to degrade colors, provide an effective substitute for traditional techniques [5]. Since the enzymatic method may be used to treat materials that are resistant to conventional methods, it may offer advantages. Additionally, according to Roriz et al. [24], it could function at both low and high pollutant concentrations throughout a wide range of salinity, pH, and temperature values. Fungal treatment of dye wastewater primarily operates through two mechanisms: biosorption, where dyes are adsorbed onto the fungal biomass, and biodegradation, where dyes are broken down by extracellular enzymes such as laccase [25]. Compared to conventional physical and chemical degradation methods, enzymatic decolorization offers significant advantages, including lower energy consumption, greater operational control, and reduced environmental toxicity [22, 26]. Laccases are of particular interest for this application due to their capacity to oxidize a broad spectrum of phenolic and non-phenolic compounds, making them highly effective agents for dye decolorization [27].

While the use of Solid-State Fermentation (SSF) and agro-industrial residues for laccase production is well-documented, this study addresses a specific, underexplored niche. The novelty lies in the systematic investigation of *Penicillium commune* S6, a fungal strain with a strong yet poorly characterized laccase system, using a specific, locally abundant, and low-valorized waste stream, Potato Peel Waste (PPW). Crucially, this study advances beyond basic substrate evaluation by employing comprehensive statistical optimization via Response Surface Methodology (RSM). This approach is applied not only to maximize enzyme yield but, more importantly, to precisely elucidate and model the complex, non-linear interactions between key physicochemical parameters, specifically pH, moisture, temperature, inoculum size, and inducer concentration, within the unique *P. commune* S6 and potato peel waste (PPW) system. By integrating strain-specific physiology with a defined agro-residue and multifactorial optimization, this work aims to develop a robust, high-yield bioprocess model with predictive power, addressing a current gap in the literature for this particular fungal strain and waste substrate combination. In this study, the screening of certain fungal isolates’ laccase-producing capacity was the first objective of the current investigation. Using 18 S RNA-based molecular techniques, the most active producer was identified, then optimizing the cultivation conditions and various media components using the conventional one factor at a time technique was achieved. Subsequently, the critical parameters will be further optimized by applying response surface methodology to obtain the appropriate production parameters and investigating their interactions. It was also discovered how well *P. commune* S6 laccase decolorized Acid Dye Lanapel Red BM 143-PL, a model dye.

## Materials and methods

### Materials

Some phenolic-rich agri-food residues that were utilized in this study were collected from the disposal of agri-food markets. The selected residues were washed thoroughly with distilled water to remove additional residues from their surface and then dried at 70 °C. We gratefully received the Acid Dye Lanapel Red BM 143-PL from the Textile Industries institute, National Research Centre, Cairo, Egypt. The other substances were all analytically graded.

### Screening of different fungal isolates for laccase secretion

Initially, different agricultural soil samples were collected, targeted the decaying plants (which are commonly possible sources of fungal laccases). In this way, a total of 15 fungal isolates were obtained from decay soil samples collected from areas contaminated with industrial waste. To isolate a new fungal laccase producer, a qualitative screening method using PDA culture medium supplemented with guaiacol (0.01%, v/v) as an inducer was conducted for the preliminary selection producers. The conversion of color to reddish-brown around the colonial fungal growth was related to guaiacol oxidation and thus as primary evidence for laccase secretion [8]. Accordingly, the positive fungal producers were investigated quantitatively using the ligninolytic production medium composed as follows (g/l): glucose, 10.5; yeast extract, 5; (NH_4_)_2_SO_4_, 2; K_2_HPO_4_, 0.5; MgSO_4_.7H_2_O, 0.5; FeSO_4_.7H_2_O, 0.02; CaHPO_4_, 0.3; ZnSO_4_, 0.2; MnSO_4_, 0.2; and CuSO_4_.5H_2_O, 0.25 [28]. The medium was adjusted to an initial pH value of 5.0 and then sterilized by autoclaving at 1.5 atmosphere and 121 °C for 15 min. Subsequently, each selected fungal producer was incubated under static and shaking conditions at 30 °C for 25 days. Therefore, the most efficient laccase producer (S6 isolate) was purified, sub-cultured, maintained at 4 °C on PDA-sterilized slants, and subjected to the identification process via molecular characterizations.

### Identification of the potent laccase-producer

The selected fungal isolate showing the highest laccase production was identified using 18 S RNA-based molecular techniques. Total genomic DNA extraction, PCR amplification, purification, and sequencing were performed using a protocol of Macrogen Company (Seoul, South Korea) (https://www.macrogen.com). The 18 S rRNA gene was amplified using the universal primers NS1 and NS8 with the sequences “GTAGTCATATGCTTGTCTC” and “TCCGCAGGTTCACCTACGGA”, respectively. The PCR was carried out under the following conditions: initial denaturation at 94 °C for 5 min, followed by 40 cycles of denaturation at 92 °C for 30 s, annealing at 54 °C for 45 s, and an extension step at 72 °C for 5 min. The amplicons were purified and then directly sequenced using the ABI 3730 DNA Analyzer (Applied Biosystems). The obtained sequences were aligned and compared with the sequences deposited in GenBank using the Basic Local Alignment Search Tool (BLAST). Molecular Evolutionary Genetic Analysis Software (MEGA version X) was used for phylogenetic analysis, and the sequences of identified phylogenetic neighbors were aligned with the sequences of representative strains using CLUSTALW. The 18 S rRNA gene sequence of the fungal strain used in this study has been deposited in the GenBank nucleotide sequence database under the accession number MT762177, and the strain was identified as *Penicillium commune* S6.

### Screening of different phenolic-rich agri-food by-products for laccase production under solid state fermentation

In this regard, *P. commune* S6 was screened for its capability to produce laccase using a low-cost culture medium based on phenolic-rich agricultural peels as a solid substrate, which was prepared and moistened (50%) with the ligninolytic production medium. Solid-state fermentation (SSF) was performed in 250-mL Erlenmeyer flasks, each containing 8–10 g of the dried phenolic-rich agricultural residue moistened to 50–60% (w/w) with the ligninolytic production medium. The flasks were plugged with cotton to allow passive aeration and incubated under static aerobic conditions, depending on the experiment. After sterilization, the solid-state culture media were inoculated with 2 ml of inoculum size of 10^7^ CFU/ml prepared under shaking conditions for 72 h of grown *P. commune* S6. Each of the inoculated residue’s media was cultivated under static conditions for 28 days at 30 °C, and laccase production was evaluated every 5 days. The extraction of enzyme was carried out in 25 mL of citrate-phosphate buffer (pH, 6; 0.1 M) under vigorous shaking conditions at 220 rpm for 30 min. Subsequently, the mixture was filtered through muslin cloth, and the resulting filtrate was used as the crude enzyme source. Finally, the preferred solid substrate that demonstrated the highest laccase induction would consequently be selected for further optimization studies.

### Laccase enzyme activity and protein estimation

The enzyme activity of laccase was evaluated using 0.5 mL of 0.3 mM ABTS solution in citrate buffer (pH 4.5, 0.1 M) as substrate and the suitably diluted enzyme sample in a total reaction volume of 2.0 ml. The absorbance increase was monitored for one minute at 436 nm using an Agilent UV-Visible spectrophotometer, and laccase activity was calculated using the following formula [29]:

$$\begin{gathered} \:Laccase\:activity\:\left( {\frac{U}{{ml}}} \right)\: = \hfill \\ \:\frac{{\Delta \:A\:\: \times \:\:{V_{t\:\:\:}} \times \:\:{{10}^6}}}{{\Delta \:t\: \times \:\:l\: \times \:\:\:\: \times \:\:{V_s}\: \times \:\:1000}} \hfill \\ \end{gathered} $$

where *l* is the cuvette diameter (1 cm), V_t_ is the total assay volume, V_s_ is the enzyme sample volume, Δ_A_ is the absorbance difference, Δ_t_ is the incubation duration (min), and ε is the ABTS extinction coefficient (ε_436_ = 29,300 M^− 1^ cm^− 1^). A unit of laccase is defined as its capacity to oxidize one µmol of ABTS per minute. Bovine serum albumin (BSA) was used as the reference protein in the Bradford technique to measure the protein content.

### One-factor-at-a-time (OFAT) optimization of *P. commune* S6 laccase production

To determine the preliminary effectiveness of each of the independent parameters and their significant range, an individual optimization procedure (one factor at a time, *OFAT*) was applied. Looking for the best solid substrate that displayed a plausible laccase-inducer, potato peel waste (PPW) was proven to be the best one. Seven different parameters, including PPW content (2.5–20 g/250 mL flask; W/V), moisture content (20–80%), CuSO_4_ concentration (1–6 mM), incubation period (10–40 day), different pH values (pH 5.0-9.5), temperatures (25–40 °C), and inoculum size (2.5–10%), were optimized.

### Optimization of medium with the response surface methodology (RSM)

Further optimization of the significant parameters was conducted using the central composite design (CCD) procedure. To boost *P. commune* S6 laccase production, the significant key parameters that notably influenced laccase production from the OFAT method were subjected to further optimization design via response surface methodology (RSM) using CCD approach. The 4-factor-5-level central composite design (CCD) with thirty experiments was performed to identify the optimal concentration and the mathematical correlation between the four important variables and the response. All four variables were investigated at low level (-1), zero level (0), and high level (+ 1), respectively, with additional -α and α values within the following ranges: (A) CuSO_4_, 0–10 mM; (B) temperature, 20–40 °C; (C) PPW, 1–20 g/250mL flask; and (D) pH value, 2.5–8.5. The second order polynomial equation was used to determine the association between various variables and the final response:

$$\:{Y={\beta\:}_{0}+\:\sum\:{\beta\:}_{i}{X}_{i}+\:\sum\:{\beta\:}_{ii}{X}_{i}^{2}+\sum\:{\beta\:}_{ij}{X}_{i}{X}_{j}\:\:}_{}$$

Where β_0_ is a constant, β_i_ is the linear effect, β_ii_ is the squared effect, and β_ij_ is the interaction effect; Y is the predicted response; and X_i_, $\:{X}_{i}^{2}$, and X_j_ are coded values for the variables used. Statistical analysis of the model was performed to evaluate the analysis of variance (ANOVA), and the quadratic models were represented as 2D contour and 3D surface plots using Design Expert^®^ (Version 7.0.0) software.

### Measurement of dye decolorization efficacy

Acid Dye Lanapel Red BM 143-PL was used as a model dye to test the *P. commune* S6 crude laccase’s capacity to decolorize dyes. Additional dilutions were used to get the necessary dye concentrations from the reserve dye aquas solution (1000 mg/L), which was stored at 4 °C without light. Based on the dye’s maximum absorption wavelength and an absorbance value of no more than 1.5, the initial dye concentrations were chosen. The experiment involved mixing 950 µL of dye solution in 0.1 M sodium citrate buffer (pH 4.5), in the presence or absence of 1 mM of Hydroxy benzotriazole (HBT) as a mediator, with 50 µL of *P. commune* S6 laccase (1.23 U/mL).

Using spectrophotometry, the decolorization process was seen as the relative reduction in absorbance at 0, 15, 30, 45, 60, 90, 120, and 180 min, respectively. It was conducted in the dark at room temperature without shaking. Using a German Agilent Carry-100 UV-Vis Spectrophotometer, the residual dye concentration was measured spectrophotometrically between 350 and 800 nm. The measurement involved computing the plot area under the peak and accounting for the potential for dye molecules to change into other structures and be absorbed at different wavelengths. The Lambert-Beer law might work since the dye samples were further diluted before testing to get absorbance values lower than 1.0 [17, 24]. The identical dye reaction combination was used for the control sample, except for the laccase solution, and the experiment settings remained the same. The following formula was used to get the decolorization percentage:

$$\begin{gathered} \:Decolorization\:\left( \% \right) \hfill \\ = \frac{{Abs{\:_{initial}} - Ab{s_{\:final}}}}{{Abs{\:_{initial}}}}\: \times \:100 \hfill \\ \end{gathered} $$

where *Abs*_*initial*_ and *Abs*_*final*_ are the total absorption area under the 350–800 nm scanning spectra at zero time and the measurement time [17].

### Analytical statistics

Except otherwise indicated, all data were from triplicate studies, and the experimental outcomes were reported as average ± standard deviation.

## Results and discussion

### Screening of different fungal isolates for laccase secretion

Primary qualitative screening for the detection of laccase-producers by the guaiacol oxidation method was carried out for 15 fungal isolates derived from decay soil samples around contaminated regions with industrial waste. Collectively, four isolates were characterized as laccase-producers, as indicated by the change in color to reddish-brown around the colonial fungal growth with varied diameter length. Accordingly, a quantitative screening method was carried out to select the potent producers among them during the 25-day incubation period. As revealed in Table 1, the ability of each of the tested fungal isolates to produce the laccase enzyme in the ligninolytic production medium was so varied that it was found to be significantly dependent on the production time during the incubation period. In this regard, we can note that the potent producer isolate S6 linearly increased laccase production during the incubation time, reaching its maximum value at the end of the cultivation period with 0.33 U/mL of activity and 0.27 U/mg of specific activity. Following this, isolates S16 and S14 demonstrated the highest enzymatic productivity at 20 d; however, a slight decrease was observed at the end of the incubation period. Meanwhile, isolate S9 observed lower laccase productivity even at 25 d. Phenols undergo one-electron oxidation, which is catalyzed by laccases and produces phenoxy radicals. These very reactive radicals create colorful oligomers and polymers by non-enzymatic polymerization. The amount of extracellular laccase released is closely correlated with the color’s intensity and distribution. Variations of the laccase induction were found to be mainly based on the nature of the fungal isolate and their ability to convert guaiacol color to reddish-brown around the colonial fungal growth, which was a primary evident for laccase production [8].

**Table 1:** Screening of the most efficient laccase-producer isolates

Isolate No.	Laccase evaluation/day											
	10			15			20			25		
	Activity (U/mL)	Protein (mg/mL)	Specific activity (U/mg)	Activity (U/mL)	Protein (mg/mL)	Specific activity (U/mg)	Activity (U/mL)	Protein (mg/mL)	Specific activity (U/mg)	Activity (U/mL)	Protein (mg/mL)	Specific activity (U/mg)
S6	0.00	1.09	0.00	0.12	1.25	0.096	0.21	1.11	0.18	0.33	1.18	0.27
S9	0.00	1.118	0.00	0.00	1.319	0.00	0.002	1.229	0.001	0.009	1.371	0.006
S16	0.00	1.79	0.00	0.007	2.11	0.003	0.18	2.17	0.08	0.11	2.68	0.04
S14	0.005	0.89	0.005	0.08	1.22	0.06	0.16	1.12	0.14	0.15	1.29	0.11

### Identification of fungal laccase-producer by 18S rRNA gene sequence analysis

**Fig. 1:**
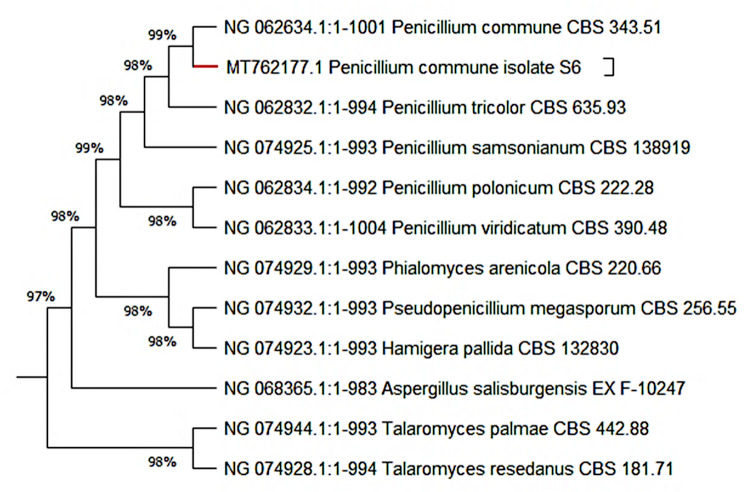
Phylogenetic tree of the potent-laccase producer

The advanced identification procedure based on the molecular characterization of isolate S6 was followed as described by Othman et al. [8]. In this way, 18S rRNA gene amplification was implemented using key primers for fungal strains. After analysis of the target sequence by the nucleotide BLAST on the NCBI, results showed the highest similarity of our isolate with *Penicillium* species, with more than 98%. Consequently, a phylogenetic tree was plotted (Fig. 1) using Mega X, which clearly demonstrated the relatedness between the highest similarities. Obviously, the highest percentage was found, corresponding to the *P. commune* strain with 99%. Thus, the morphological and molecular observations confirmed that the isolate S6 consistently belonged to the *P. commune* strain and was designated as *P. commune* S6. In addition, deposition of this strain in GenBank under accession number MT762177 was carried out.

### Screening of different phenolic-rich agri-food residues for laccase production under solid-state fermentation

The ability of the potent strain *P. commune* S6 to secrete laccase enzyme under solid-state conditions was examined using different solid agri-food wastes as compared to that produced by standard culture medium under submerged conditions. As shown in Table 2, a considerable yield of laccase was detected using potato peel waste (PPW), which was increased 2.5-fold (0.68 U/mg) when compared to the control (0.27 U/mg). In addition, a notable inductivity of laccase production using banana peels (BP) (0.695 U/mg) and apple peels (AP) (0.414 U/mg) was obtained after 21 days of incubation, emphasizing the efficiency of these substrates to enhance laccase production. Nevertheless, other residue substrates showed an inability to encourage laccase induction by *P. commune* S6. Indeed, the growth and production rate of *P. commune* S6 proved there was no proportional relationship between them. Despite the few differences between PPW and BP as substrates for enzymatic induction, PPW was chosen as a potent inducer substrate to complete further optimization studies due to economic and environmental considerations over BP. Investigation of different phenolic-rich agri-food residues (PRAR) to be displayed as a major substrate for the improvement of laccase yield by *P. commune* S6 could indicate a significant productivity increase if compared to the synthetic culture medium, which increased by 2.55-fold. These naturally occurring phenolic chemicals function as intricate, multifaceted inducers. They mimic the lignocellulosic environment that the fungus naturally inhabits, indicating the existence of a substrate (lignin) that needs laccase to break down. Compared to a single synthetic molecule, this induction signal is more powerful and comprehensive [30]. In accordance with our results, PPW, AP, and BP are promising attractive substrates for laccase induction due to their high carbohydrate and phenolic content [31]. A significant laccase yield by *P. commune* S6 using these food-processing wastes could be attributed to the abundance of phenolic compounds involved in these wastes.

**Table 2:** Screening of laccase production via solid state fermentation using agriculture wastes

Substrate	Laccase/ day ABTS assay Specific activity (U/mg)			
	7	14	21	28
Control (broth medium) (U/mL)	0.006	0.012	0.011	0.27
Potato peels (U/gds)	0	0.019	0.362	0.687
Banana peels (U/gds)	0	0.006	0.695	0.548
Apple Peels (U/gds)	0	0.421	0.414	0.317

### Laccase optimization via OFAT technique

**Fig. 2:**
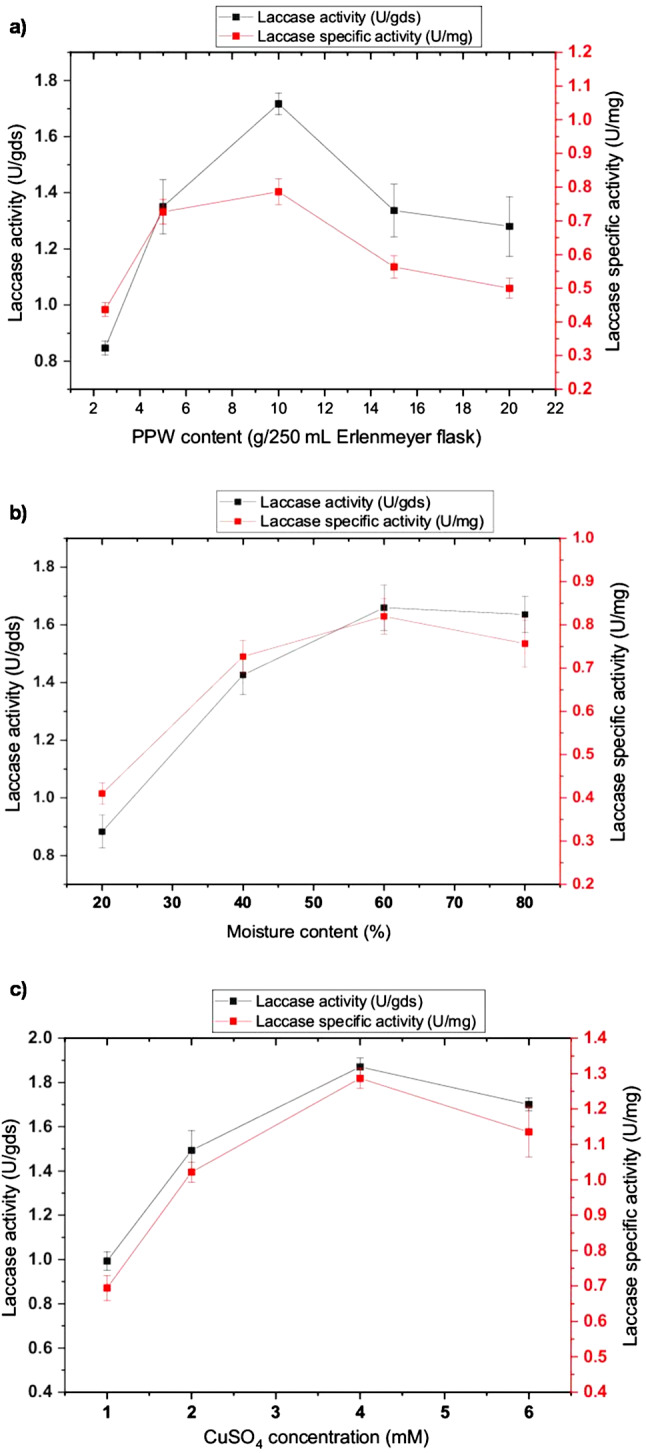
Effect of **a**) PPW content, **b**) Moisture content, and **c**) CuSO_4_ concentration on laccase production

The effects of different cultural conditions that can help define the effective parameters toward enhancing enzymatic productivity were studied under solid-state fermentation. The influence of the substrate content on the laccase yield showed that the weight of solid substrate induced the enzymatic productivity at 40 g/L; an increase or decrease in PPW weight about this weight was correlated with a notable decrease in the enzymatic productivity (Fig. 2a). In addition, the effect of the PPW moistened ratio demonstrated that the highest laccase productivity (0.78 U/mg) was obtained when the solid substrate was moistened with 60% (Fig. 2b). These findings demonstrate the crucial and nonlinear impact that moisture content plays in solid-state fermentation for the synthesis of laccase. Below 40%, there is a distinct lower threshold where insufficient hydration significantly limits microbial metabolism and substrate accessibility, leading to a sharp 50% reduction in enzyme production. On the other hand, because of effective oxygen diffusion via the substrate matrix, the system exhibits a remarkable tolerance to high moisture, sustaining excellent output even at 80%. The existence of a clear upper moisture boundary for maximizing laccase biosynthesis is highlighted by the fact that exceeding this tolerance limit causes a decline because too much water fills pore spaces, resulting in anaerobic conditions and decreasing gas exchange.

**Fig. 3:**
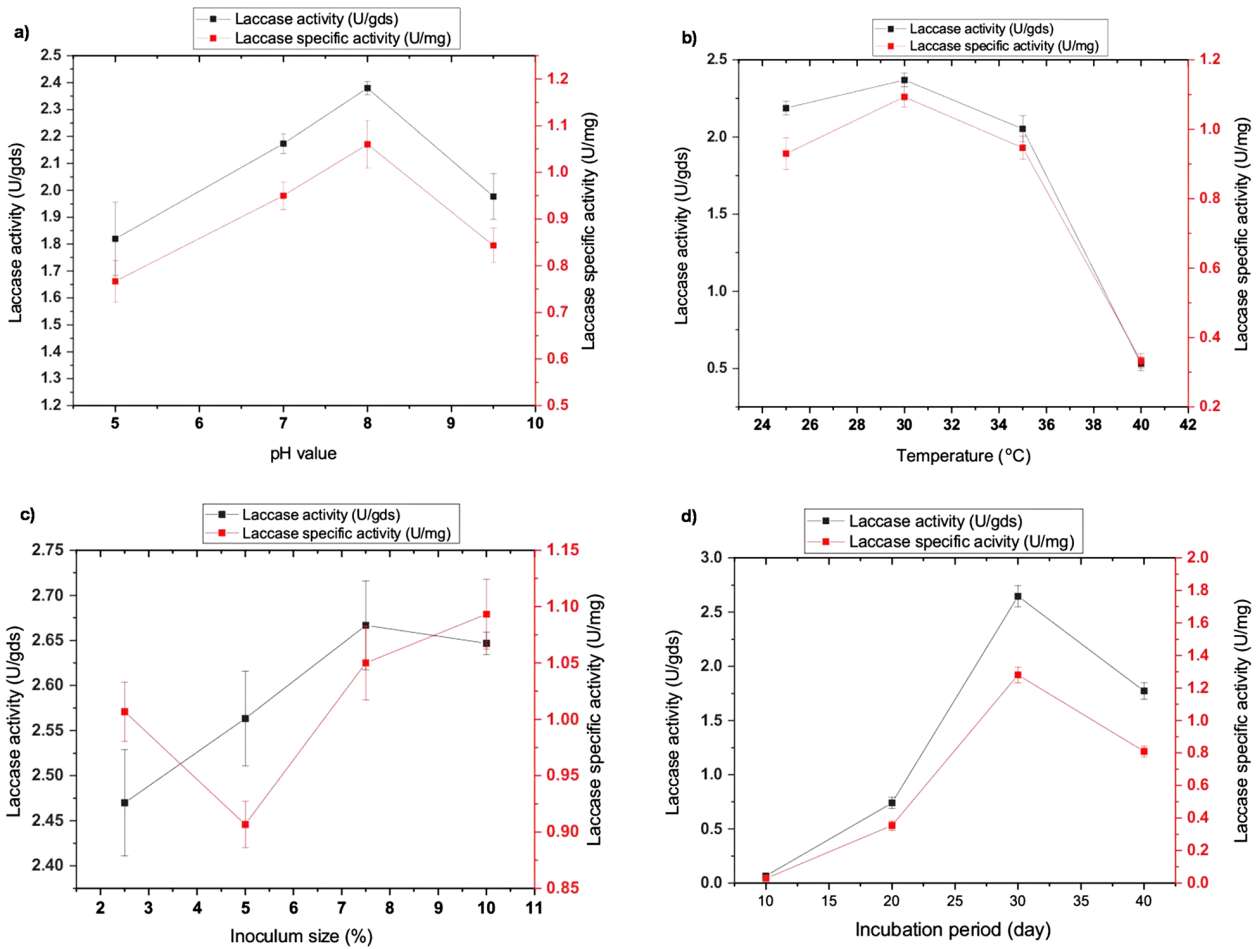
Effect of **a**) pH value, **b**) temperature, **c**) inoculum size, and **d**) incubation period on laccase production

Incorporating copper ions into the cultural medium can greatly promote laccase yield. Thus, different concentrations of CuSO_4_ were tested for their capability of enhancing laccase productivity. It was observed that all concentrations had an inducible effect on laccase secretion, which reached its highest value at 4 mM. enzymatic productivity started to decline as copper concentration increased, which became toxic for fungal growth, resulting in a significant reduction in laccase production (Fig. 2c). These results demonstrate copper’s dual and concentration-dependent function in laccase production as a vital cofactor and a strong metabolic inhibitor. With optimal secretion reached at 4 mM, which probably corresponds to the saturation of the enzyme’s active sites and maximal transcriptional activation, the universal inductive effect across all tested concentrations validates that copper functions as a fundamental molecular signal for laccase gene expression. The ensuing drop in productivity, however, marks a dramatic shift from induction to toxicity, where high copper levels interfere with vital cellular functions, reduce fungal viability, and eventually reduce enzyme yield. This highlights the fine balance between nutrient supply and metabolic stress in fungal biotechnology. On the other hand, the different pH values of the PPW cultural medium were investigated from 5.0 to 9.5. The main effect of pH was found in the neutral and alkaline regions, where the laccase yield showed a gradual increase, reaching the optimum value (1.08 U/mg) at pH 8.0. With further increases in the pH level, the production curve decreased. Moreover, laccase production was significantly influenced by the acidic pH values, which were lower than those produced in alkaline medium with 34.3% (Fig. 3a). This preference implies that the isolate has laccase isoforms with stability and activity profiles tailored to neutral-alkaline environments, maybe to take advantage of certain substrate properties or ecological niches within the waste stream. The fall beyond pH 8.0 could be due to the fact that excessive alkalinity probably starts to compromise the integrity of fungal membranes, change the solubility of nutrients, or inactivate the released enzyme itself.

Otherwise, laccase yield by *P. commune* S6 was not affected by the incubation temperature factor; the maximum productivity was obtained at 30 °C. However, higher temperatures than 35 °C caused a severe inhibition of both fungal growth and enzyme production. Besides, lower temperatures were observed to cause a slight decrease in enzymatic productivity (Fig. 3b). This suggests a strong physiological adaptation, as the fungus’ secretory and metabolic systems function well in typical mesophilic environments. But there are definite limits to this resilience: temperatures above 35 °C cause extreme heat stress, which interferes with cellular functions and impairs both growth and enzyme synthesis. Likewise, the inoculum concentration of *P. commune* S6 was found to have a slight effect on the laccase yield. Since the differences in productivity between the higher and lower inoculum sizes are small, the maximum productivity was found at 7% of the inoculum amount (1.09 U/mg); a slight reduction in enzymatic yield was detectable with further increases or decreases (Fig. 3c). The appearance of an optimum at 7% indicates that a balance is required: enough biomass to quickly colonize the substrate and start the production of enzymes, but not so much as to overcrowd and prevent productive secondary metabolism or premature nutrient depletion. On the thirtieth day, the enzyme production peaked, and after that, the rate at which the enzyme was produced began to progressively decrease (Fig. 3d). Our findings support other research, which shows that the ideal incubation period for fungus to produce maximum laccase production might range from 7 to 36 days [32]. While enzyme secretion may occur during the primary growth phase, it typically peaks during the idiophase and is triggered by the depletion of carbon or nitrogen supplies [8, 17].

### Enhancement of *P. commune* S6 laccase synthesis with RSM-CCD

Optimizing fermentation processes using effective techniques to produce appropriate and reasonably priced enzymes is a critical duty in the enzyme industry. Statistical methods that identify the crucial variables and their interactions might be used to achieve this [8, 33]. RSM is a popular approach to experiment design that makes use of statistics to determine the influence and relationships between a few selected parameters on a given response [34]. The best circumstances for high titer *P. commune S6* laccase production were determined by applying the RSM-CCD technique, which was also utilized to investigate the effects of interactions between certain physiological and nutritional parameters [8, 17].

To find the most successful ones, several researchers frequently optimized the independent factors utilizing the OFAT approach before performing the advanced statistical analysis [35]. Using peach waste (PW) and solid-state fermentation, the culture conditions for *Pleurotus eryngii’s* production of laccases were optimized [36]. Similarly, orange peel waste was used in central composite design (CCD) to optimize laccase synthesis by *Pleurotus pulmonarius* during solid state fermentation [37]. Additionally, *Aspergillus niger* was used to produce laccase under solid state conditions from wheat straw, and the greatest laccase productivity was obtained at 2.551 U/mL following statistical optimization [38]. The culture medium for laccase production by *Pleurotus sajor-caju* was improved using experimental design of RSM and established under solid state fermentation utilizing wheat straw [39].

The results of the OFAT experiments indicated that A-CuSO_4_, B-incubation duration, C-PPW concentration, and D-pH were the main parameters impacting the generation of laccase by *P. commune S6*. Therefore, precise optimization of these efficient components was necessary for a potential optimization course. We used an RSM-CCD experiment for that goal, which was based on findings on the optimization of laccase production by *P. commune S6* using the OFAT approach. The explored range of the selected parameters (CuSO_4_, incubation duration, PPW concentration, and pH) was established based on the obtained OFAT data.

Based on the results of OFAT testing, five levels (-α, -1, 0, + 1, + α) were developed for each parameter that was selected. An investigation form was constructed by assessing actual laccase activities from the thirty trials of the generated CCD throughout RSM in order to determine the ideal production response (Table 3). The quadratic model was used to justify the statistical relationship between the independent components and the dependent answer. The CCD data on the link between *P. commune S6* laccase development and the four factors that were examined—A, B, C, and D—were explained by the following coded variables equation. In terms of factors that are coded, the last equation is:

**Table 3:** Central composite design (CCD) of the selected parameters and their response

Run	CuSO_4_ conc. (mM)	Incubation period (d)	PPW content (g/250 ml flask)	pH	Laccase activity (U/gds)	
					Actual	Predicted
1	2.5	35	15.25	4.0	2.053	1.943
2	7.5	35	15.25	7.0	3.681	4.195
3	2.5	25	5.75	7.0	3.044	3.279
4	5	30	10.5	5.5	3.922	4.165
5	5	30	1.0	5.5	1.633	2.590
6	7.5	35	5.75	7.0	3.252	3.503
7	7.5	35	5.75	4.0	1.779	1.650
8	5	40	10.5	5.5	4.045	4.332
9	5	30	10.5	5.5	4.299	4.165
10	5	30	10.5	5.5	4.401	4.165
11	2.5	35	5.75	4.0	2.005	1.686
12	5	30	10.5	5.5	4.244	4.165
13	7.5	25	5.75	4.0	1.223	1.318
14	2.5	25	5.75	4.0	1.755	0.915
15	5	30	10.5	8.5	5.077	4.897
16	5	20	10.5	5.5	3.411	3.179
17	7.5	25	15.25	4.0	1.386	2.108
18	7.5	25	5.75	7.0	2.822	2.606
19	7.5	35	15.25	4.0	1.889	1.925
20	10	30	10.5	5.5	2.205	1.246
21	5	30	20	5.5	4.955	4.053
22	5	30	10.5	5.5	4.311	4.165
23	2.5	25	15.25	7.0	4.662	4.466
24	2.5	35	5.75	7.0	5.662	4.615
25	5	30	10.5	2.5	0.028	0.264
26	5	30	10.5	5.5	3.811	4.165
27	2.5	25	15.25	4.0	1.667	1.686
28	0	30	10.5	5.5	0.922	1.936
29	7.5	25	15.25	7.0	3.222	3.812
30	2.5	35	15.25	7.0	5.112	5.288

$$\begin{gathered} \:Laccase\:activity\: = \:4.16\: - \:0.173\:A\: + \:0.288\:B\: \hfill \\ + \:0.366\:C\: + \:1.158\:D\: - \:0.110\:A\:B\: + \:0.005\:A\:C \hfill \\ \: - \:0.269\:A\:D\: - \:0.129\:B\:C\: + \:0.141\:B\:D\: + \:0.104\:C\:D \hfill \\ \: - \:0.643\:{A^2}\: - \:0.102\:{B^2}\: - \:0.211\:{C^2}\: - \:0.396\:{D^2} \hfill \\ \end{gathered}$$

where A, B, C, and D represent the coded values for CuSO_4_, incubation time, PPW content, and pH, respectively. The model’s F-value of 0.707 suggests that there is only a 0.02% chance that the model’s F-value may be the result of noise, which supports the model’s significance as shown by the ANOVA analysis of variance (Table 4). Model terms are deemed significant only when the “Prob > F” values are less than 0.05. Important parameters in the present model are C, D, A^2^, and D^2^. The resulting model’s quadratic terms A² (CuSO_4_ conc.) and D² (pH) are significant not just statistically but also because they provide light on the physiological and biochemical constraints controlling *P. commune* S6’s laccase synthesis. The significant, concave-downward parabolic connection between copper concentration and enzyme yield is indicated by the coefficient for the A² term (*p* < 0.001). This provides strong evidence that the determined optimal copper level is a real maximum. The sharp drop on either side of this peak emphasizes copper’s dual function: at ideal concentrations, it is a necessary cofactor and inducer, but at greater concentrations, it becomes a metabolic inhibitor, probably interfering with cellular processes and causing metal toxicity to the fungus. Similarly, a distinct alkaline optimum is defined by the substantial D² term (*p* < 0.05) for pH. The yield peak and subsequent drop are explained by this statistically proven quadratic effect, highlighting the fact that laccase stability and fungal metabolism are precisely adjusted to a certain pH window. Nutrient intake, the effectiveness of enzyme secretion, or the catalytic stability of the laccase isoforms themselves are probably all negatively impacted by deviations from this optimum. On the other hand, words with “Prob > F” values greater than 0.10 in the model are deemed unimportant. With “Adeq Precision,” the signal/noise ratio is calculated; a number greater than 4.0 indicates a desirable ratio. The current model’s “Adeq Precision” ratio of 10.049 indicates a strong signal, suggesting that the model can effectively navigate the design space. A diagnostic study of the produced RSM for laccase synthesis by *P. commune S6* was conducted using residual analysis. The residual analysis offers further information about the developed RSM model. The impacts of the residuals that affect laccase production were investigated using a normal probability plot [40]. A regularly distributed residual population was suggested by the straight line that was formed at every position in the normal plot of residuals vs. the internally studentized residuals (Fig. 4a), which showed the distribution of the residuals. Moreover, this suggests a high degree of agreement between the actual experimental results and the laccase production levels predicted by the model (Fig. 4b). Data is normalized and altered using a statistical method known as the Box-Cox transformation in order to approximate a reliable, workable model. The Box-Cox transformation is used to enhance the quadratic model results and residual normality by selecting a suitable exponent (Lambda = λ). The Lambda number indicates the power to which all data should be enhanced [8, 41]. The Box-Cox power transformation plot for laccase production optimization in the current quadratic model is displayed in Fig. 4c. It illustrates how the usual distribution of residuals is impacted by power transformation. These results suggest that the developed model may be utilized to identify the optimal circumstances under which the *P. commune S6* isolate would manufacture laccase.

**Table 4:** Analysis of variance (ANOVA) table for response surface quadratic model

Source	Sum of squares	df	Mean square	F value	*P*-value (Prob > F)
Model	54.390	14	3.885	7.771	0.0002
A-CuSO_4_	0.714	1	0.714	1.429	0.2506
B-Incubation time	1.995	1	1.995	3.991	0.0642
C-PPW content	3.208	1	3.208	6.416	0.0230
D-pH	32.197	1	32.197	64.405	< 0.0001
AB	0.193	1	0.193	0.386	0.5440
AC	0.0003	1	0.0003	0.0007	0.9795
AD	1.156	1	1.156	2.312	0.1492
BC	0.264	1	0.264	0.528	0.4784
BD	0.320	1	0.320	0.640	0.4363
CD	0.173	1	0.173	0.346	0.5650
A^2^	11.354	1	11.354	22.712	0.0003
B^2^	0.287	1	0.287	0.574	0.4605
C^2^	1.218	1	1.218	2.437	0.1393
D^2^	4.304	1	4.304	8.610	0.0103
Residual	7.499	15	0.499		
Lack of Fit	7.213	10	0.721	12.629	0.0059
Pure Error	0.286	5	0.057		
Cor Total	61.889	29			

Std. Dev., 0.707; R-Squared, 0.879; Adj R-Squared, 0.766; Adeq Precision, 10.049

**Fig. 4:**
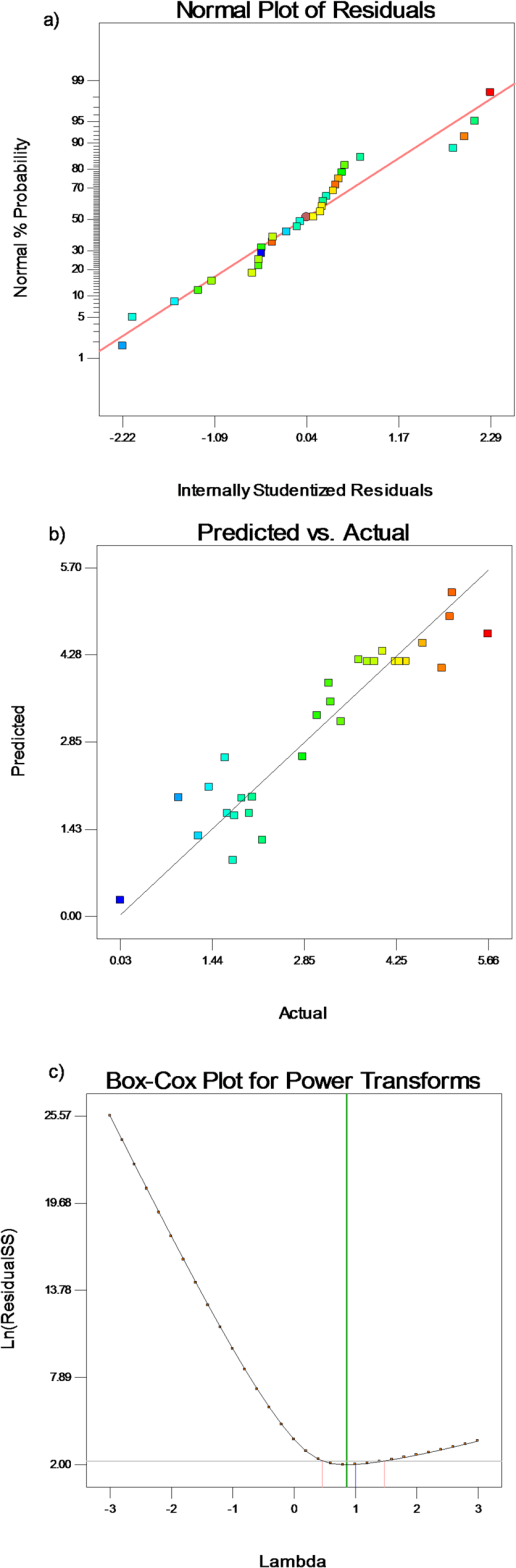
*P. commune* S6 laccase production diagnostic analysis of the generated RSM: (**a**) the normalized residuals; (**b**) the predicted vs. actual values; and (**c**) the power transforms Box-Cox plot

**Fig. 5:**
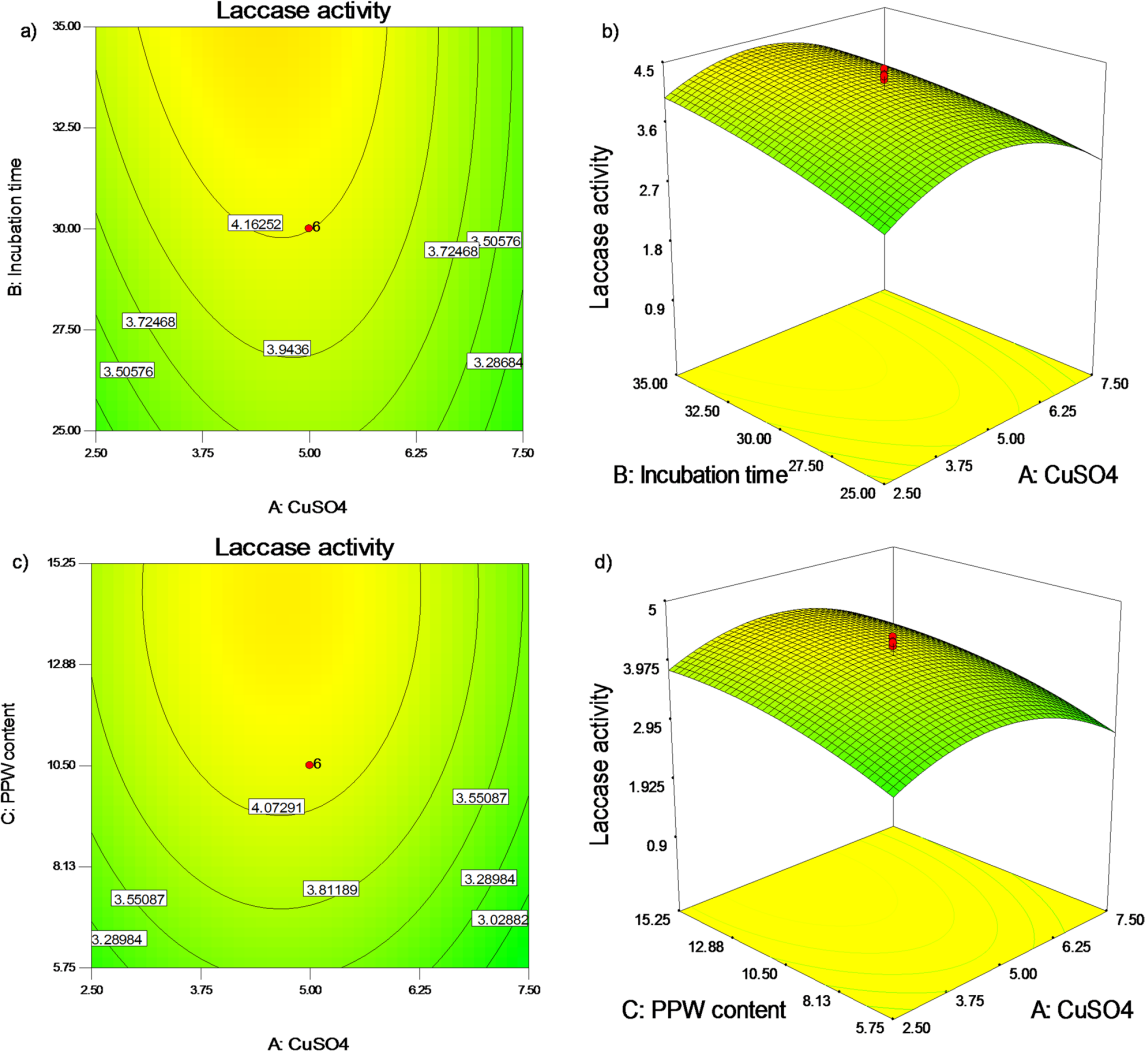
2D counter and 3D surface plots of interactions between (**a**,** b**) CuSO_4_ concentrations and incubation period, and (**c**,** d**) PPW content and CuSO_4_ concentrations on formation of *P. commune S6* laccase at the midpoints of the other two parameters in each case

**Fig. 6:**
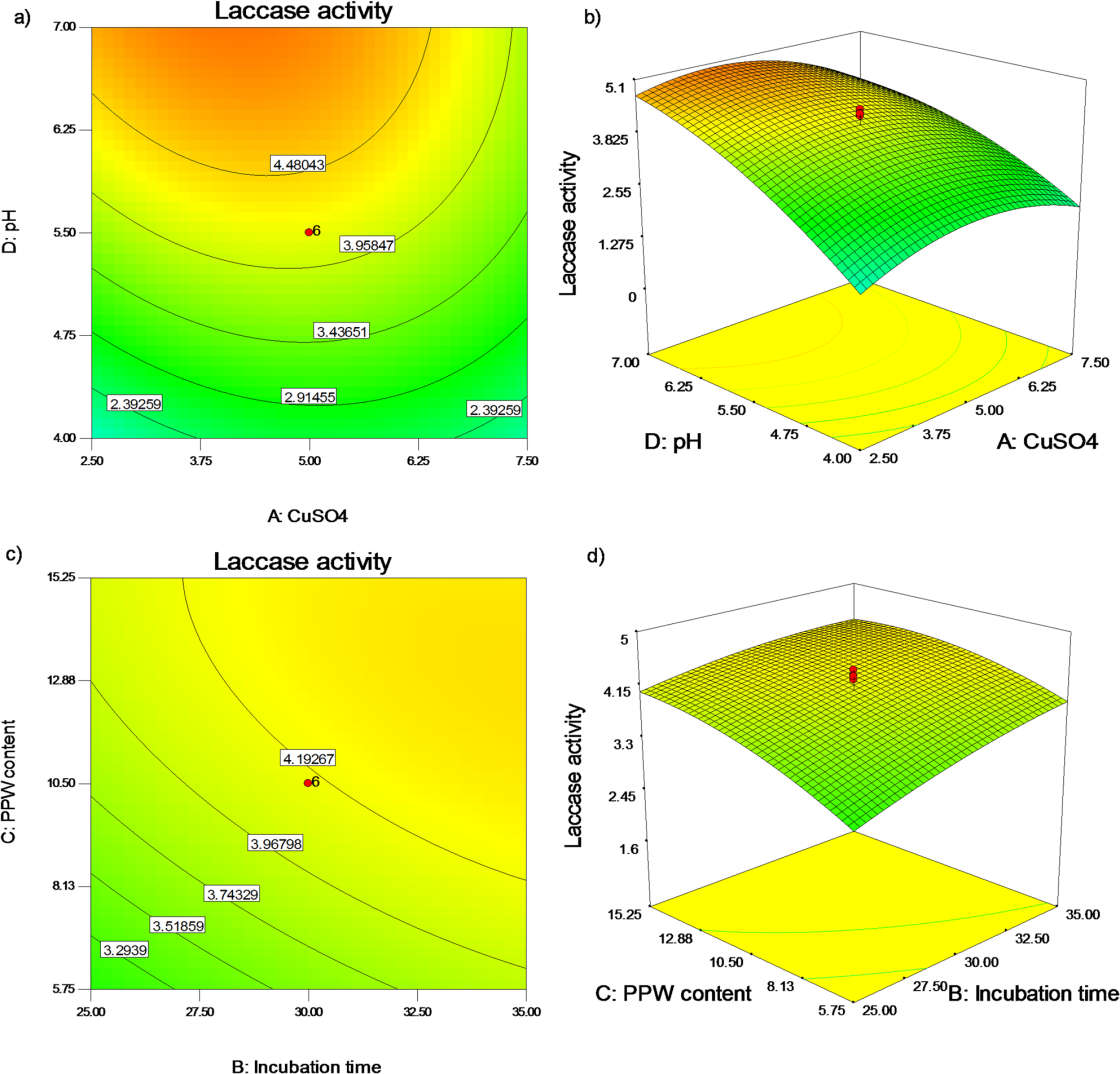
2D counter and 3D surface plots of interactions between (**a**,** b**) CuSO_4_ concentrations and pH values, and (**c**,** d**) PPW content and incubation period on formation of *P. commune S6* laccase at the midpoints of the other two parameters in each case

**Fig. 7:**
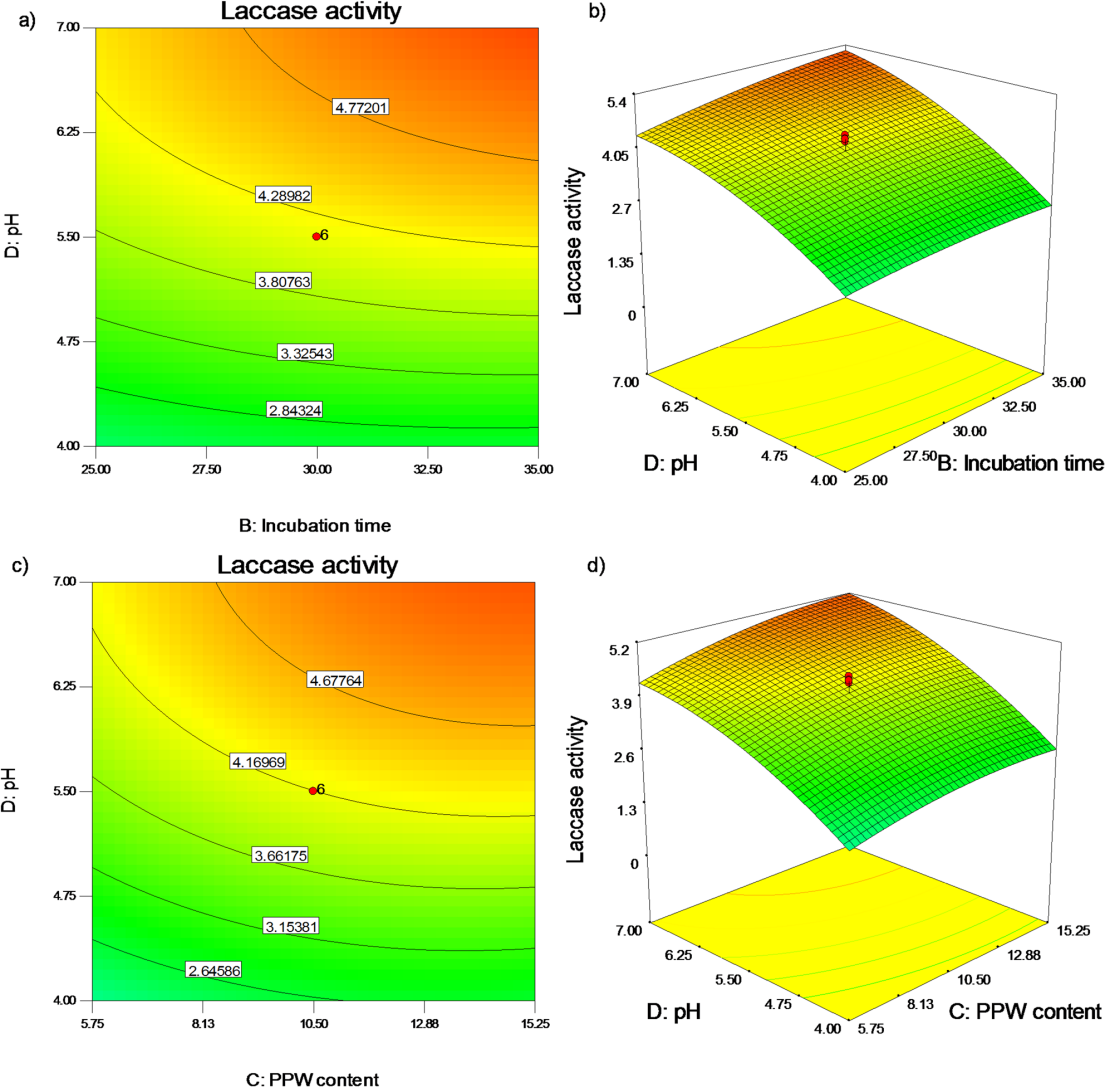
2D counter and 3D surface plots of interactions between (**a, b**) pH values and incubation period, and (**c, d**) PPW content and pH values on formation of *P. commune* S6 laccase at the midpoints of the other two parameters in each case

The 2D counter and 3D surface plots in Figs. 5, 6 and 7 demonstrated how the conditions under investigation impacted *P. commune* S6 laccase formation. When investigating values that differ from those of the other two components, two of the four parameters—the pH value of 5.5 (D), the PPW content of 10.5 g/flask (C), the CuSO_4_ value of 5 mM (A), and the incubation period of 30 days (B)—were fixed at their center values to create the presented graphs. Figures 5a, b illustrate how different CuSO_4_ concentrations (A), and incubation periods (B) affect *P. commune* S6 laccase production (the response) at a constant PPW level of 10.5 g/flask (C), and pH value of 5.5 (D). Laccase production rises when CuSO_4_ concentration gradually rises to 5.0 mM and subsequently decreases, while laccase formation rises as incubation time increases (Fig. 5a, b). The effects of varying PPW content (C) and CuSO_4_ concentrations (A) in the production medium on *P. commune* S6 laccase synthesis throughout a 30-day predefined incubation period (B) and pH of 5.5 (D) are shown in Fig. 5c, d. The response design that was discovered indicates that laccase production rises in proportion to an increase in CuSO_4_ concentration up to 5.0 mM. Conversely, a higher PPW concentration in the production medium considerably increases laccase production.

The effects of the interaction between CuSO_4_ concentrations (A) and pH value (D) at fixed values of 10.5 g of PPW content per flask (C) and 30 days of incubation time (B) are displayed in Fig. 6a, b. The response curve that was produced indicates that laccase production rose steadily as pH value (D) increased and peaked at pH 7.0, even though the ideal CuSO_4_ concentration was around 5.0 mM (Fig. 6a, b). In contrast to our findings, Mathur et al. [42] optimized the development of *Coriolus sp.* laccase utilizing the Plackette-Burman design and were able to obtain a 6.5-fold increase in laccase productivity at pH 5.0. Competent *Trametes sp*. LS-10 C laccase production was produced by Li et al. [43] at pH 4.0 in a more acidic region using RSM. The effects of PPW content (C), and incubation time (B), at fixed values of pH value 5.5 (D), and CuSO_4_ value of 5 mM (A) were plotted in Fig. 6c, d. The plot shows how the two parameters have a direct impact on laccase synthesis, which increases until the concentrations of both parameters reach 15.25 g/flask after 35 days of incubation (Fig. 6c, d).

Additionally, Fig. 7a, b illustrated how the synthesis of *P. commune* S6 laccase was impacted by pH value (D) and incubation period (B) at the predefined midpoints of CuSO_4_ value of 5 mM (A) and PPW content of 10.5 g/flask (C). The benefits of maintaining both parameters at high values for 35 days at pH 7.0 are shown in the three-dimensional plot. Finally, the interactions between the pH value (D) and the PPW content (C) at constant concentrations of 5 mM CuSO_4_ (A) and after 30 days of incubation (B) are shown in Fig. 7c, d. As seen by the surface plot, high values of both parameters up to 15.25 g/flask and pH 7.0 increased laccase production by *P. commune* S6 (Fig. 7c, d). Model predictions state that laccase production would be 6.20 U/gds at 2.71 mM CuSO_4_, 46 days of incubation, 12.64 g/flask of PPW content, and pH 9.13, accordingly (Fig. S1, S2). Table S1 presents comparative laccase activity values based on optimized process parameters for laccase production at each step of the study.

### Acid dye Lanapel red BM 143-PL decolorization by *P. commune* S6 laccase

**Fig. 8:**
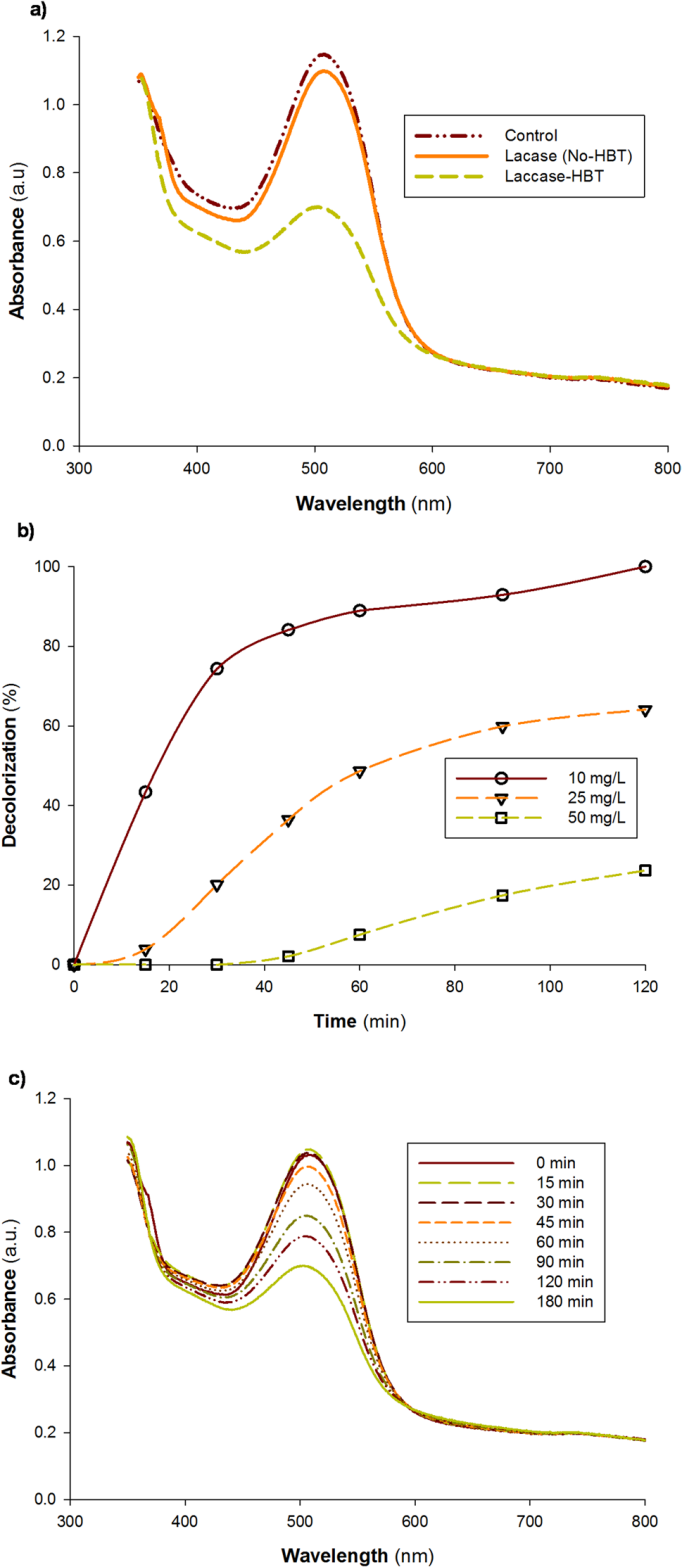
Decolorization of Acid Dye Lanapel Red BM 143-PL by *P. commune* S6 laccase. **a**) effect of HBT (1 mM) as a redox mediator with laccase after 180 min using 1.23 U of enzyme and 50 mg/L of dye, **b**) The decolorization of various dye concentrations over time using 50 µL of laccase (1.23 U/mL) and 1mM HBT, **c**) The dye decolorization absorption spectrum at 50 mg/L when redox mediators are present. After treating Acid Dye Lanapel Red BM 143-PL (50 mg/L) with 50 µL of laccase (1.23 U/mL) for several incubation times in the presence of 1 mM HBT as a mediator, scanning spectra were obtained

The ability of different laccases from different sources to decolorize dyes has been varied [27, 44]. Acid Dye Lanapel Red BM 143-PL is extensively used in different industries around the world, and it is considered as one of the major wastewaters pollutants. Acid Dye Lanapel Red BM 143-PL was chosen to be decolorized in current study by *P. commune* S6 laccase in presence and absence of redox mediators. The results obtained declared the difference in decolorization ability of the *P. commune* S6 laccase in the presence or absence of HBT as a redox mediator toward Acid Dye Lanapel Red BM 143-PL degradation (Fig. 8a), where the decolorization efficiency was clearly affected by the presence of the redox mediator (HBT), and almost no decolorization was conducted in the absence of HBT, which declare the dependency of *P. commune* S6 laccase on redox mediators to oxidize Acid Dye Lanapel Red BM 143-PL. *P. commune* S6 laccase was found to be able to decolorize the Acid Dye Lanapel Red BM 143-PL by 88.92, 48.64 and 10.64% at concentrations of 10, 25, and 50 mg L^− 1^ after 60 min and by 100, 64.08, and 36.86% after 120 min, respectively (Fig. 8b). The dye decolorization absorption spectra at 10, 25, 50, 100, and 200 mg/L of Acid Dye Lanapel Red BM 143-PL by *P. commune* S6 laccase in the presence of 1 mM HBT as a mediator were presented in Fig. S3. No absorbance peaks should be found after decolorization as a sign of high-performing dye wastewater treatment [17, 22]. The *P. commune* S6 laccase enzyme system is extremely promising for Acid Dye Lanapel Red BM 143-PL decolorization, based on the results of the current case study, which showed a high decolorization outcome. With no further absorbance peaks at the concentration of 10 mg/L throughout the visible spectrum after 120 min, Fig. S3a demonstrated that the visible spectrum of the Acid Dye Lanapel Red BM 143-PL decolorization could be verified based on the absorbance drop between 350 and 800 nm. It is worth mentioning that no decolorization of Acid Dye Lanapel Red BM 143-PL was detected in control samples without enzyme. Scanning spectra for Acid Dye Lanapel Red BM 143-PL solution (50 mg·L^− 1^) decolorization by *P. commune* S6 laccase were recorded at different reaction times in the presence of HBT as a redox mediator. The significant variation in decolorization effectiveness clearly shows that the laccase from *P. commune* S6 is strictly dependent on a synthetic redox mediator such as HBT and cannot directly oxidize the complex Acid Dye Lanapel Red BM 143-PL. The enzyme’s oxidation potential is inadequate to attack the dye’s structure in the absence of HBT, leading to little decolorization. When HBT is present, a catalytic cycle is created in which the laccase oxidizes the tiny mediator molecule, which diffuses and oxidizes the bulky, non-phenolic dye via electron shuttling, significantly expanding the scope of the reaction. This mediator-dependency is a crucial functional feature that determines the enzyme’s appropriateness for uses involving stubborn synthetic dyes and directs the necessity of mediator addition in industrial bioremediation procedures. The operation of a Laccase-Mediator System (LMS) via a Long-Range Electron Transfer (LRET) mechanism is strongly supported by the dramatic activity in the presence of HBT. During this cycle, HBT is oxidized by the laccase to its nitroxyl radical, which diffuses to the dye and serves as a diffusible electron shuttle. The radical starts a series of events that eventually rupture the chromophore and cause decolorization by abstracting an electron, most likely from the dye’s electron-rich azo bond (–N = N–) or an associated amine group. The results declared that the decolorization process is a time dependent process, and decolorization efficiency is forward proportional to the reaction time between the enzyme and dye (Fig. 8c).

## Conclusions

This study successfully established *Penicillium commune* S6 as a potent laccase producer using potato peel waste (PPW) as a sustainable cultivation medium. Through sequential optimization via one-factor-at-a-time (OFAT) and Response Surface Methodology (RSM) with a Central Composite Design (CCD), laccase secretion was significantly enhanced. The RSM model identified CuSO₄ concentration, incubation time, PPW content, and pH as the critical, interacting parameters governing production. Statistical validation via ANOVA confirmed the model’s high significance and reliability for navigating the design space. The optimized conditions, 2.71 mM CuSO₄, 46 days incubation, 12.64 g PPW per flask, and pH 9.13, predicted a maximum laccase yield of 6.20 U/gds. Furthermore, the crude laccase demonstrated high efficiency in dye bioremediation, achieving complete (100%) decolorization of Acid Dye Lanapel Red BM 143-PL at 10 mg L⁻¹ within 120 min in the presence of redox mediators, with substantial removal at higher concentrations (64.08% at 25 mg L⁻¹ and 36.86% at 50 mg L⁻¹). These findings highlight the dual advantage of the developed process: it valorizes an agricultural residue into a low-cost medium and produces a robust enzymatic preparation effective under mild conditions. Consequently, *P. commune* S6 laccase presents a promising, eco-friendly biocatalyst for applications in textile effluent decolorization and other industrial bioprocesses where reduced chemical and energy inputs are desirable.

## Supplementary Information

Below is the link to the electronic supplementary material.

Supplementary Material 1

## Data Availability

The 18 S rRNA gene sequence of the fungal strain used in this study has been deposited in the GenBank nucleotide sequence database under the accession number MT762177.

## Abbreviations

OFAT: One factor at a time
BLAST: Basic Local Alignment Search Tool
CCD: Central composite design ()
PRAR: Phenolic-rich agri-food residues
HBT: Hydroxy benzotriazole
ANOVA: The analysis of variance
PPW: Potato peel waste
