# Supplementary material for: Boosting laccase production by Penicillium commune s6 via solid-state fermentation on phenolic agri-wastes: statistical enhancement and bioremediation application in dye decolorization
Source: BMC Biotechnol. 2026 Mar 11;26:42. doi: 10.1186/s12896-026-01108-2 (PMC13063652; doi:10.1186/s12896-026-01108-2)
Supplement: Supplementary file 1 — Supplementary Material 1 [file 12896_2026_1108_MOESM1_ESM.docx]

**Supplementary materials:**

Table S1: A comparative table based on optimizing process parameters for laccase production

Fig. S1: The ramp model for laccase optimization desirability.

Fig. S2: The predicted model for laccase optimization desirability.

Fig. S3: The dye decolorization absorption spectra at 10, 25, 50, 100, and 200 mg/L of acid dye Lanapel Red BM 143-PL by *P. commune* S6 laccase. After treating acid dye Lanapel Red BM 143-PL with 50 μL of laccase (1.23 U/mL) for several incubation times in the presence of 1 mM HBT as a mediator.

Table S1: A comparative table based on optimizing process parameters for laccase production

| **Parameter** | **Laccase activity (U/gds)** | **Specific activity (U/mg)** |
| --- | --- | --- |
| Screening (Agri-wastes; PPW) | 0.16 | 0.687 |
| PPW content | 1.70 | 0.78 |
| Moisture content | 1.70 | 0.82 |
| CuSO_4_ conc | 1.85 | 1.27 |
| pH value | 2.40 | 1.08 |
| Temperature | 2.40 | 1.10 |
| Inoculum size | 2.75 | 1.09 |
| Incubation time | 2.75 | 1.30 |
| RSM Design | 5.29 |  |
| RSM Optimization | 6.2 |  |

**Supplementary figures**

Fig. S1: The ramp model for laccase optimization desirability.

Fig. S2: The predicted model for laccase optimization desirability.

| a) |  |
| --- | --- |
| b) |  |
| c) |  |
| d) |  |
| e) |  |

Fig. S3: The dye decolorization absorption spectra at 10, 25, 50, 100, and 200 mg/L of acid dye Lanapel Red BM 143-PL by *P. commune* S6 laccase. After treating acid dye Lanapel Red BM 143-PL with 50 μL of laccase (1.23 U/mL) for several incubations in the presence of 1 mM HBT as a mediator.
